# A Case of Growth Hormone Use in Dyggve–Melchior–Clausen Syndrome

**DOI:** 10.1155/2022/8542281

**Published:** 2022-03-15

**Authors:** Ravi Upadhyay, Claire Ruane, Rachel Umans, Beth A. Pletcher, Aditi Khokhar, Kristin Wong

**Affiliations:** ^1^Departments of Medicine and Pediatrics, Rutgers University New Jersey Medical School, Newark, NJ, USA; ^2^Department of Pediatrics, Rutgers University New Jersey Medical School, Newark, NJ, USA

## Abstract

Short stature has many causes including genetic disease, skeletal dysplasias, endocrinopathies, familial short stature, and nutritional deficiencies. Recombinant growth hormone (rGH) therapy may be employed to improve stature based on the underlying etiology and growth velocity. Skeletal dysplasia in Dyggve–Melchior–Clausen (DMC) syndrome tends to be progressive, typically with hip involvement, and ultimately leads to bilateral dislocation of the hip joints. Here, we present a pediatric patient with short stature treated with rGH therapy, complicated by the development of debilitating, bilateral hip pain, and found to have DMC syndrome. Our patient had limited range of motion at several joints including the hips after receiving 6 months of rGH therapy. Given the timing of the patient's rGH therapy and the progression of her disease, it is difficult to determine if there were any benefits and instead, is concerning for worsening of her skeletal dysplasia with rGH therapy use. Consequently, patients with severe short stature should have a thorough workup for genetic causes like DMC syndrome, before initiating rGH therapy to determine any potential benefits or harms of treatment.

## 1. Introduction

Short stature is defined as a height of 2 standard deviations (SDs) or more below the mean height for children of that age and sex in a given population. Extreme cases of short stature, such as height standard deviations below 2.5 or 3, account for approximately 0.6 and 0.1% of the general population, respectively, and are usually associated with syndromic conditions such as skeletal dysplasias [1]. After a thorough workup, she was diagnosed with Dyggve–Melchior–Clausen (DMC) syndrome, a rare autosomal recessive genetic disorder. This syndrome is associated with short stature, intellectual disability, and a host of skeletal abnormalities which can affect the hips, vertebrae, and extremities [2]. Short stature is a universal feature of the disease with presentation typically after 5 years of age [3]. Recombinant growth hormone (rGH) therapy may be considered in patients with certain genetic diseases and idiopathic short stature, but in patients with severe short statures, especially those with skeletal disproportion and other developmental abnormalities, a thorough workup, including investigation of possible endocrine and genetic causes, is warranted to identify any underlying condition that should preclude treatment. In this case report, we present a pediatric patient who had progressive, severe bilateral hip pain after treatment of her short stature with rGH.

## 2. Case Presentation

A 12-year-old Hispanic girl, with a past medical history of short stature and microcephaly, presented to the ambulatory clinic to establish care. Physically, her mother reported that the patient had always been “small for her age.” She was noted to have growth delays and was followed by pediatric endocrinology beginning at 7 years of age in Honduras. At age 10.5 years, she was started on rGH therapy (dose unknown). Therapy was discontinued after 6 months on noting a disproportionate growth of limbs relative to the torso. She also reported that the patient had severe pain in the bilateral thighs, knees, and posterior calves with inability to walk. The pain gradually worsened over the prior four months and was also associated with a regression in her gross motor skills, including her ability to jump and ride a bike. The patient's gross motor milestones were otherwise appropriate until her recent difficulties ambulating.

Developmentally, the patient began to exhibit speech and language delays beginning at the age of 18 months. She was not able to use 2-word phrases until 4 or 5 years of age. She also reported a history of fine motor delays, being unable to tie her shoes or print her name. Her emotional and cognitive abilities were also delayed and the patient had never learned to name colors, recite the alphabet, or participate in elaborate play.

Birth history revealed that the patient was born full-term via a repeat cesarean section to nonconsanguineous Honduran parents after an uneventful pregnancy. There were no complications during delivery; the patient was noted to have microcephaly at birth, but no other abnormalities were identified. The mother was only able to reproduce a viral test report showing Rubeola IgG+ and IgM−. Upon social history, the patient had immigrated from Honduras 5 months prior to presentation and had no other significant exposures or risks. Her family history was also noncontributory to the patient's condition, and she had two healthy siblings. There was no family history of learning or intellectual disabilities nor neurological, growth, or genetic disorders.

Physical examination on presentation revealed a short (height 121.9 cm, <1%, *Z* = −3.80; weight 30.8 kg, 5%, *Z* = −1.63; BMI 20.75 kg/m^2^, 80%, *Z* = 0.83; head circumference 49.5 cm, < 1%; midparental height 160 cm, 6%) and an alert, social, and friendly girl who looked younger than the stated age. Her vital signs were within normal limits for age. At 12 years, she was able to speak in simple phrases and name some objects but was unable to name letters or numbers, count fingers, or write her name. She was unable to answer simple questions and could follow only very simple commands. She had a short neck, broad chest, kyphosis with no scoliosis, brachydactyly with broad feet and hands, and Tanner 2 breast development. Lower extremity exam revealed severe genu valgum, tenderness over bilateral posteromedial knees, and generalized, diffuse lower extremity tenderness, with a limited active range of motion due to pain; no pain elicited on passive movement. Bilateral hip flexor strength was 4/5 (limited by pain), while it was 5/5 in all the other major muscle groups. Clumsy fine motor movements were noted in the upper extremities with normal tone, intact sensation, and normal reflexes. No spasticity, atrophy, tremors, ataxia, or dysmetria were found. Her gait was notable for genu valgum, intoeing, and asymmetric step with limping greater on the right than the left side. She was able to take only a few steps without support.

**Figure 1:**
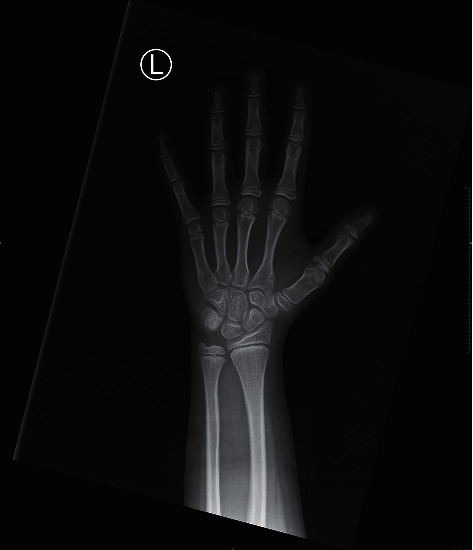
PA radiograph of the left hand for bone age evaluation. A radiograph of the left hand is depicted. The patient's chronological age at the time of the evaluation was 12 years and 2 months. According to the second edition of Greulich and Pyle, the patient's bone age is 11 years. Also noted is a short first metacarpal bone. The remaining bones have normal morphology. There is normal bone mineral density.

**Figure 2:**
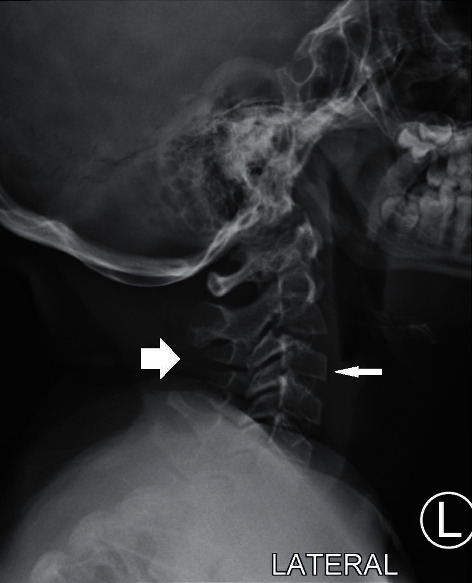
The cervical spine visualized to the C5 level. The vertebrae are diminished in height and dysmorphic in appearance (thick arrow). There is anterior wedging (thin arrow).

**Figure 3:**
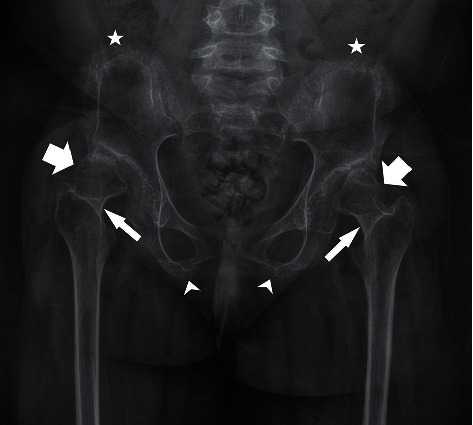
Pelvic and hip abnormalities associated with DMC syndrome. The pelvis with both hips is depicted. Both femoral heads are essentially dislocated/severely subluxated from the shallow, dysplastic acetabulum with resultant formation of a pseudoacetabulum superior and lateral to the native acetabulum bilaterally (thick arrow). Both femoral heads are deformed and small in size (thick arrow). There is premature growth plate fusion at the proximal femoral epiphyses (thin arrow). The iliac rest margins appear deformed (star). The ischial bones and inferior pubic rami are hypoplastic as well (arrowhead). There is decreased bone mineral density throughout, and the bones appear gracile.

The patient was referred to genetics, endocrinology, neurology, developmental pediatrics, and orthopedics. Laboratory workup including thyroid-stimulating hormone, lead level, hepatitis panel, and human immunodeficiency virus testing was all normal (Table 1). Her bone age was consistent with her chronological age. Her chromosomal microarray, however, showed normal dosage with a single large region of homozygosity on the long arm of chromosome 18, which increased concerns for an autosomal recessive condition. Radiographic imaging revealed a shortened 1st metacarpal bone of the left hand, diminished height of the cervical vertebrae along with a dysmorphic appearance (Figures 1 and 2), exaggerated lumbar lordosis, severe subluxation of bilateral femoral heads with dysplastic acetabulae and pseudoacetabulum formation (Figure 3), deformed iliac crests with hypoplasia of inferior pubic rami (Figure 3), and bowing of the right femur. Magnetic resonance imaging of the brain depicted partial agenesis of the corpus callosum and distal body of the splenium but was otherwise unremarkable. Subsequent genetic testing included an Invitae Skeletal Dysplasia Panel which revealed a homozygous missense mutation in a highly conserved region of the DYM gene (c 1244A⟶T, p.His415Leu in exon 11), with algorithms predicting this to be a pathogenic mutation. This particular mutation has not been seen in population databases but has been seen in patients with DMC syndrome. Written informed consent for publication of this case report was provided by the parent.

## 3. Discussion

Our case revolves around a 12-year-old girl with short stature, developmental delays, and skeletal dysplasia who was eventually diagnosed with DMC syndrome and had bilateral femoral head subluxation which was preceded by a 6-month course of rGH therapy in Honduras. DMC syndrome is a rare progressive genetic disorder which is characterized by intellectual disability, microcephaly, short stature, and progressive spondyloepimetaphyseal dysplasia, with skeletal deformities such as shallow acetabular roofs, lace-like iliac crests, and small femoral heads [3]. Approximately 100 cases have been described in the literature to date. The genetic syndrome is caused by mutations in the dymeclin (DYM) gene on the long arm of chromosome 18 which encodes a protein that is necessary for normal skeletal development and brain function [4]. The exact mechanism of the osteochondrodysplasia requires further elucidation, but DYM deficient murine models suggest that disorganized chondrocyte ossification is the result of impaired vesicular trafficking to and from the Golgi complex [5].

According to one study, age at presentation can vary with a reported range of 3 to 17 years, although delays in motor and intellectual development tend to be identified in the first few years of life [3]. The very specific lace-like appearance of the iliac crests and double-humped vertebral bodies become evident by 3-4 years of age [6]. Interestingly, one study found that subluxation of the hips is not a universal feature of the disease [3]. When present, progressive hip deformities in patients with DMC syndrome invariably lead to bilateral hip subluxation and eventual bilateral dislocation of the hip joint. The pain associated with changes at the hip is ultimately managed surgically although this can be delayed until late adolescence in some cases [7]. At the time of our initial evaluation, our patient already had limited range of motion at several joints including the hips. Radiographic studies revealed severe subluxation at the hips bilaterally along with bowing of the right femur. Our patient falls within the reported age range for skeletal dysplasia progression described in patients with DMC syndrome, but it is not known if prior rGH therapy could have precipitated or worsened hip subluxation in this case given the close timing of events.

Histochemical analysis of iliac crest growth plates in patients with DMC syndrome has revealed that a defect in endochondral ossification may cause skeletal dysplasia [8, 9]. Studies thus far have not evaluated the GH axis in patients with this syndrome, and the role of rGH therapy in this disorder has not been studied either. Our patient received daily rGH injections for six months before it was self-discontinued by the family on noting disproportionate limb growth, and she presented within a year of treatment with debilitating hip pain and gait abnormalities. To the best of our knowledge, this is the first reported case of rGH therapy use in a patient with DMC syndrome.

It is interesting to note that rGH therapy in other syndromes with skeletal dysplasias have had mixed results. On occasion, DMC syndrome can be misdiagnosed as a mucopolysaccharidosis (MPS) due to similar radiologic and clinical features [10]. Recombinant growth hormone therapy has shown some benefit in patients with MPS with reversion of growth deceleration, although controlled clinical trials are lacking [11, 12]. One clinical trial demonstrated efficacy of rGH therapy in patients with achondroplasia with only modest clinical impact, but not in patients with endochondral ossification abnormalities, such as spondyloepiphyseal dysplasia congenita, a disorder that shares features of skeletal dysplasia with DMC disease [13]. Therefore, it is difficult to ascertain whether rGH treatment was of any benefit to our patient, or if it accentuated the patient's skeletal dysplasia or precipitated her hip subluxation. Recombinant growth hormone therapy has indeed been associated with several orthopedic complications including scoliosis and slipped capital femoral epiphysis [14]. Adult height is severely reduced in DMC syndrome with a height range of 115–127 cm [15]. It will be interesting to see what the postpubertal height of this patient will be and whether it will be outside of the range of final height typically seen in this disorder. In our patient with severe short stature, skeletal disproportion, and developmental abnormalities, a thorough genetic workup was likely indicated prior to initiation of rGH therapy. Further studies are needed involving patients with DMC in order to determine the indications of use and possible adverse effects of rGH therapy in this rare genetic disorder.

## Figures and Tables

**Table 1:** Laboratory analysis.

Laboratory results on presentation	
Thyroid stimulating hormone	3.4 uu/ml
Free T4	1.0 ng/dl
Parathyroid hormone	31 pg/ml
Calcitriol	54.7 pg/ml
ESR	17 mm/hr
CRP	<1 mg/l
Lead	<1 ug/dl
HIV antibody/antigen screen	Nonreactive
Phosphorus	4.3 mg/dl
Alkaline phosphatase	233 u/l
*Complete blood count*	
White blood cell count	8.4 × 10^3^/ul
Hemoglobin	13.7 g/dl
Hematocrit	41.1%
Platelet count	298 × 10^3^/ul
Creatinine kinase	126 u/l

## Data Availability

No data were used to support this study.
